# Impact on predictability of tropical and mid-latitude cyclones by extra Arctic observations

**DOI:** 10.1038/s41598-018-30594-4

**Published:** 2018-08-14

**Authors:** Kazutoshi Sato, Jun Inoue, Akira Yamazaki, Joo-Hong Kim, Alexander Makshtas, Vasilli Kustov, Marion Maturilli, Klaus Dethloff

**Affiliations:** 1 National Institute of Polar Research, Tachikawa, 190-8518 Japan; 2 Kitami Institute of Technology, Kitami, 090-8507 Japan; 3 Application Laboratory, Japan Agency for Marine-Earth Science and Technology, Yokohama, 236-0001 Japan; 4 SOKENDAI (Graduate University for Advanced Studies), Hayama, 240-0193 Japan; 5 Korea Polar Research Institute, Incheon, 21990 Korea; 6 Arctic and Antarctic Research Institute, Sankt-Peterburg, 199397 Russia; 7 Alfred Wegener Institute, Helmholtz Centre for Polar and Marine Research, Potsdam, 14473 Germany

## Abstract

Recent research has demonstrated that additional winter radiosonde observations in Arctic regions enhance the predictability of mid-latitude weather extremes by reducing uncertainty in the flow of localised tropopause polar vortices. The impacts of additional Arctic observations during summer are usually confined to high latitudes and they are difficult to realize at mid-latitudes because of the limited scale of localised tropopause polar vortices. However, in certain climatic states, the jet stream can intrude remarkably into the mid-latitudes, even in summer; thus, additional Arctic observations might improve analysis validity and forecast skill for summer atmospheric circulations over the Northern Hemisphere. This study examined such cases that occurred in 2016 by focusing on the prediction of the intensity and track of tropical cyclones (TCs) over the North Atlantic and North Pacific, because TCs are representative of extreme weather in summer. The predictabilities of three TCs were found influenced by additional Arctic observations. Comparisons with ensemble reanalysis data revealed that large errors propagate from the data-sparse Arctic into the mid-latitudes, together with high-potential-vorticity air. Ensemble forecast experiments with different reanalysis data confirmed that additional Arctic observations sometimes improve the initial conditions of upper-level troposphere circulations.

## Introduction

The forecast skill of tropical cyclone (TC) tracks has improved substantially in recent decades, extending the TC forecast period to five days^1,2^. This improved predictability has been attributed to the advance of both numerical weather prediction models^3–5^ and data assimilation techniques^6^, although major errors persist in certain cases. Other factors (e.g., improved initial fields with enhanced observation data) would improve TC track forecasts^6–8^. Several field experiments have released dropsondes into TC environments (e.g., Dropwindsonde Observations for Typhoon Surveillance near the Taiwan Region^3,9^, the Observing System Research and Predictability Experiment – Pacific Asian Regional Campaign^6,8^, and synoptic surveillance missions with a Gulfstream IV-SP jet aircraft^10^) to enhance the skill of TC forecasts. Assimilated additional dropsonde observations near TC centres in the upper troposphere have reduced uncertainty in reanalysis data^7^, improving the prediction of TC tracks^6–8^. Observing system experiments (OSEs) have shown that dropsonde observations in sensitive regions, depicted by a singular vector method^11^, have strong impact on TC location forecasts in comparison with observations made outside such regions^7,8^. However, when a TC reaches the mid-latitudes, its motion becomes sensitive to the present upper-troposphere circulations^12^.

Arctic observations, which improve troposphere circulation representation at upper levels, have strong potential for improving TC forecasts in the extratropics. There is considerable error and uncertainty in reanalysis data over the Arctic Ocean, not only because the logistics and harsh environments restrict the number of stations and their observation frequency^13^ but also because Arctic atmospheric circulations are difficult to model^14–16^. Although satellite observations with higher resolution and frequency are useful for improving the reproducibility of atmospheric circulations over the Arctic^17^, reanalysis data have substantial uncertainties and biases (errors) at high latitudes, particularly near the pole and the surface of the ice and ocean^18^. Such errors and uncertainties are incorporated in the analysis fields used in operational numerical weather prediction systems as initial conditions^13^. Therefore, additional Arctic radiosonde observations might reduce the uncertainty and errors in the analyses^18,19^, improving the prediction of atmospheric circulations over the Arctic^20–22^. Localised potential vorticity (PV) anomalies, often called “tropopause polar vortices”, have been shown to play a role in surface cyclone development over the Arctic Ocean^23,24^, sometimes extending into the mid-latitudes because of large meandering jet streams at the fringe of the tropospheric vortex^25^ (as during summer 2016). Geopotential height anomalies over East Asia in August 2016 and over the Atlantic Ocean in September 2016 showed extensive jet meanders. Therefore, it is expected that improvement in the reproducibility of atmospheric circulations at high latitudes would contribute to improved accuracy of severe weather forecasts for the mid-latitudes, even in summer.

## Observations and cyclones in August and September 2016

**Figure 1:**
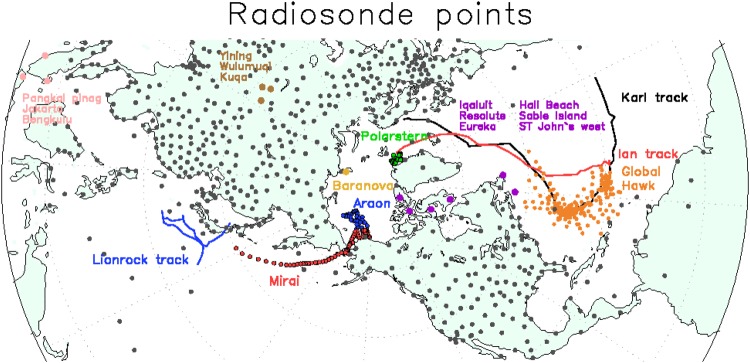
Radiosonde observation sites during August and September 2016. Dots show radiosonde observation sites at land stations (gray), Cape Baranova (yellow), NASA Global Hawk (orange), RVs *Araon* (blue), *Polarstern* (green), and *Mira*i (red), Sable Island, Resolute, Iqaluit, Hall Beach, ST John’s west, and Eureka (purple), Yining, Wulumuqi, and Kuqa (brown), and Pangkal Pinang, Jakarta, and Bengkulu (pink). Blue, red and black lines show tracks of Typhoon Lionrock, Tropical Storms Ian and Karl. Grid Analysis and Display System (GrADS) version 2.0.2 (http://cola.gmu.edu/grads/) was used to create the map in this figure.

To investigate the impact of additional observations on the predictability of weather patterns at mid-latitudes, special radiosonde observations from ships and land-based stations were conducted over the Arctic Ocean during August and September 2016 (Fig. 1). The Japanese RV *Mirai* conducted an Arctic cruise in the Chukchi and Beaufort seas during 1–22 September 2016. Most of the radiosonde data were sent to the Global Telecommunication System in real time (Supplementary Fig. 1a), presumably reducing uncertainties in atmospheric fields of the reanalysis data and improving the initial conditions for operational weather forecasts. The North Atlantic Waveguide and Downstream Impact Experiment^26^ ran from 19 September until 16 October 2016, increasing the number of radiosonde observations from Canadian stations to 4 times a day (Fig. 1 and Supplementary Fig. 1b). Additional radiosonde observations were made at 0600 and 1800 UTC. In addition, dropsonde observations were taken over the North Atlantic during 20 and 25 September using the National Aeronautics and Space Administration (NASA) Global Hawk unmanned aircraft to improve track forecasts for Tropical storm Karl (Fig. 1). The dropsonde data were assimilated into operational weather forecast systems (Supplementary Fig. 1c). In August, twice-daily radiosonde observations were conducted during the Arctic cruises of both the Korean RV *Araon* in the Chukchi and East Siberian seas and the German RV *Polarstern* in the Fram Strait (Supplementary Fig. 1d). During the same period, observations at the Russian land station at Cape Baranova were made once per day (79.3°N, 101.8°E). However, radiosonde observation datasets from the Baranova land station and the cruises were not sent to the Global Telecommunication System, which meant they were not used in operational weather forecast systems.

We conducted ensemble data assimilation and ensemble forecast experiments to estimate the impact of observations on the representation of and forecast skill for mid-latitude atmospheric circulations. For the OSEs (data denial experiments), multiple different data-assimilation streams composed of repeated data-assimilation-forecast cycles with different observations (CTL and all OSEs) were prepared for the two periods of August and September 2016, that is the September and August streams (see Methods). For the September stream, we created CTL_1_, OSE_MGC,_ OSE_M_, OSE_G_, OSE_C_ reanalysis datasets from 15 August to 28 September 2016. CTL_1_ included observations from the RV *Mirai*, NASA Global Hawk^27^, and Canadian stations. The OSE_MGC_ comprised the additional radiosonde data that were removed from CTL_1_. Three reanalysis data sets (OSE_M_, OSE_G_, OSE_C_) that excluded the additional radiosonde observation data from individual station (RV Mirai, Global Hawk and Canadian stations). For the August stream, reanalysis datasets of CTL_2_, OSE_BAP_, OSE_B_, OSE_A_, OSE_P_, OSE_MID_, OSE_TRO_ were created from 26 July to 30 August 2016. CTL_2_ included not only the observational data in the PREPBUFR global observation datasets but also additional radiosonde data from the Cape Baranova land station and RVs *Araon* and *Polarstern*. In contrast, OSE_BAP_ excluded these data. Five reanalysis datasets (OSE_G_, OSE_C_, OSE_B_, OSE_A_, OSE_P_, OSE_MID_, OSE_TRO_) that excluded the additional radiosonde observation data from individual stations (i.e., Cape Baranova, RV *Araon*, RV *Polarstern*, Midlatitude stations and Tropical stations). The observation data are assimilated in a 6-hours window by the LETKF. Details of the radiosonde data included in the CTLs and OSEs are shown in Table 1. Note that each DA forecast-analysis cycle was performed in the identical settings for all DA experiments. We conducted forecast experiments using these reanalysis datasets as initial condition.

**Table 1:** Radiosonde data used (circles) and not used (crosses) in reanalyses and forecasts.

	Mirai (24 AUG-27 SEP)	Canadian Stations (18SEP-10OCT)	Global Hawk (20–25 SEP)	Mid-latitude stations (01–31 AUG)	Tropical stations (01–31 AUG)	Araon (06–22 AUG)	Baranova (01–31 AUG)	Polarstern (01–31 AUG)
CTL_1_	◯	◯	◯	◯	◯			
OSE_MGC_	×	×	×	◯	◯			
OSE_M_	×	◯	◯	◯	◯			
OSE_G_	◯	◯	×	◯	◯			
OSE_C_	◯	×	◯	◯	◯			
CTL_2_	◯			◯	◯	◯	◯	◯
OSE_BAP_	◯			◯	◯	×	×	×
OSE_A_	◯			◯	◯	×	◯	◯
OSE_B_	◯			◯	◯	◯	×	◯
OSE_P_	◯			◯	◯	◯	◯	×
OSE_MID_	◯			×	◯	◯	◯	◯
OSE_TRO_	◯			◯	×	◯	◯	◯

Members in gray column indicate experiments for Karl case.

In summer 2016, there were upper-level troughs related to the tropospheric polar vortex with strong winds over the North Atlantic and East Asia, which would have influenced the TC locations in the mid-latitudes (Supplementary Figs 2, 3, and 4). Over the North Atlantic, a low pressure system developed into tropical storm Ian^28^ on 12 September. Ian was absorbed by an extratropical cyclone over the east coast of North America on 16 September (hereafter, this TC is referred to as Ian throughout its lifetime), reaching the Greenland Sea on 18 September (Supplementary Fig. 3a). In addition, near the western coast of Africa on 15 September, a tropical depression grew into tropical storm Karl, which moved northward off the east coast of North America^27^ (Supplementary Fig. 2a). Karl merged into an extratropical cyclone on 26 September (hereafter, this TC is referred to as Karl throughout its lifetime) and then propagated rapidly to reach the western coast of Norway by 28 September. A trough with a strong PV anomaly over the North Atlantic influenced the locations of Karl and Ian in their merger stages (squares in Supplementary Figs 2a and 3a). The trough with strong winds corresponded to a southward intrusion of strong PV air near the western Arctic over the Chukchi Sea. It took about a week for this air to reach the North Atlantic sector (Supplementary Fig. 5a,b).

One month before the Karl event, a TC developed into Typhoon Lionrock to the southeast of Japan on 17 August 2016, moving southwestward (Supplementary Fig. 4a). On 25 August, Lionrock started moving northward and it crossed northern Japan on 30 August. The typhoon killed 22 people in the Hokkaido and Tohoku regions of Japan and it damaged crops in the former region. A trough at the 300-hPa level with strong winds extended above the western part of Lionrock at 1200 UTC 29 August, probably influencing its northward movement on 30 August. We found that this trough, with a strong PV anomaly, originated in the Arctic Ocean on 22 August, reaching East Asia within a week when the forecast model had modest skill^29^ (Supplementary Fig. 5c).

## Impact of extra Arctic radiosonde observations on tropical and mid-latitude cyclones

**Figure 2:**
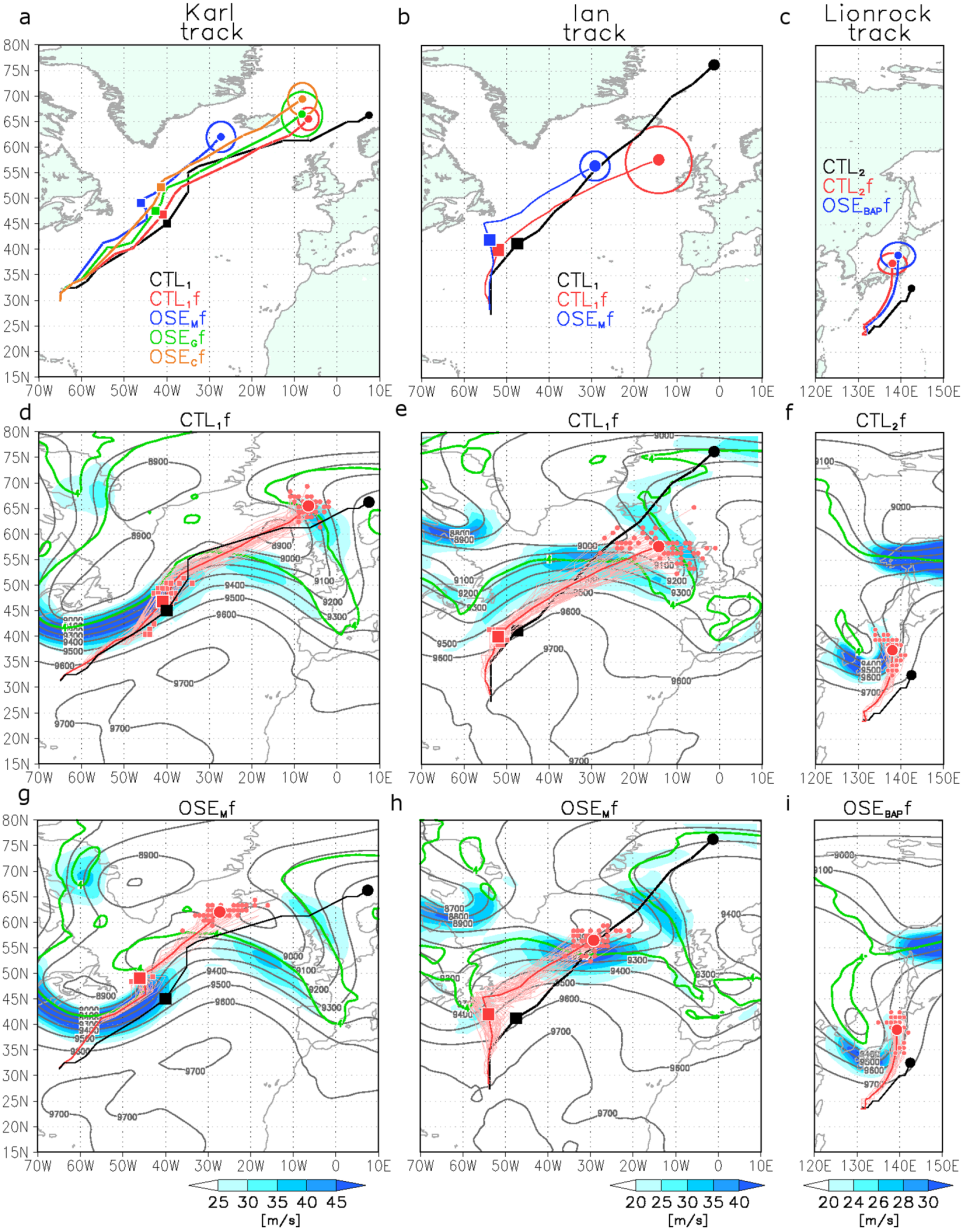
Karl, Ian, and Lionrock track forecasts. (**a**) Black line shows track of Karl from 0000 UTC 24 September through 1200 UTC 28 September in CTL_1_. Lines show ensemble mean TC tracks predicted by CTL_1_f (red line), OSE_M_f (blue line), OSE_C_f (orange line), and OSE_G_f (green line). Squares show location of Karl in merging stage with extratropical cyclone at 0000 UTC 26 September (day 2.0 forecast). (**b**) As in (**a**), but Ian tracks from 0000 UTC 14 September through 1200 UTC 18 September by CTL_1_ (black line), CTL_1_f (red line), and OSE_M_f (blue line). Squares show location of Ian in merging stage with extratropical cyclone at 0000 UTC 16 September (day 2.0 forecast). (**c**) As in (**a**), but Lionrock tracks from 0000 UTC 25 August through 1200 UTC 29 August by CTL_2_ (black line), CTL_2_f (red line), and OSE_BAP_f (blue line). Circles indicate ensemble spread. The centres of circles show locations of ensemble mean. Circle radius indicates average difference in distance between locations of ensemble mean and of each member. Predicted upper-level geostrophic wind speed (300–500 hPa), Z300 (black contour), and PV on 330 K surface (green line) at 0000 UTC 26 September 2016 in CTL_1_f (**d**), OSE_M_f (**g**), at 0000 UTC 16 September 2016 in CTL_1_f (**e**), OSE_M_f (**h**) and at 1200 UTC 29 August 2016 in CTL_2_f (**f**), OSE_BAP_f (**i**). Black and red lines show tracks of Karl from 0000 UTC 24 September through 1200 UTC 28 September in CTL_1_f (**d**) and OSE_M_f (**g**), tracks of Ian from 0000 UTC 14 September through 1200 UTC 18 September in CTL_1_f (**e**) and OSE_M_f (**h**), and tracks of Lionrock from 0000 UTC 25 August through 1200 UTC 29 August in CTL_2_f (**f**) and OSE_BAP_f (**i**) for all ensemble member. Grid Analysis and Display System (GrADS) version 2.0.2 (http://cola.gmu.edu/grads/) was used to create the maps in this figure.

To assess the impact of radiosonde observations on the forecast skill of TCs, forecast experiments (hereafter, CTLsf and OSEsf) were conducted using the CTLs and OSEs as their respective initial conditions. Figure 2a shows predicted Karl tracks from a 4.5-day forecast initialized by ensemble CTL_1_, OSE_M_, OSE_G_, and OSE_C_ analyses at 0000 UTC 24 September (forecasts, CTL_1_f, OSE_M_f, OSE_G_f, and OSE_C_f, respectively). CTL_1_f captured the observed location of Karl, whereas OSE_M_f tended to predict a slower eastward movement compared with CTL_1_f (dots in Fig. 2a,d). However, considerable difference developed in the track of Karl after it merged with the other extratropical cyclone (day 2.0 forecast, squares in Fig. 2a), and this difference amplified with forecast time (e.g., the day 4.5 forecast) (Fig. 2a). Southwesterly winds around the trough in CTL_1_f produced eastward movement of the cyclone in all members (Fig. 2d). In OSE_M_f, predicted winds were weaker than in CTL_1_f, owing to a failure to predict a southward protrusion of the trough over Newfoundland on day 2.0 (Fig. 2g and Supplementary Fig. 2e). This slowed eastward movement of the cyclone in OSE_M_f. To measure forecast skill for that trough, anomaly correlation coefficients (ACCs) were calculated for 300-hPa geopotential height fields (Z300) over North Atlantic Ocean (Supplementary Fig. 6a). The ACCs of both CTL_1_f and OSE_M_f dropped below 0.9 at the 4.0-day forecast but the ensemble have similar spread (CTL_1_f: from 0.86 to 0.92, OSE_M_f: from 0.82 to 0.88), indicating that the ACCs did not reveal the impact of extra Arctic observations on the predictability of Karl. However, the decrease of the central pressure of Karl was predicted well in CTL_1_f (Supplementary Fig. 7a,b), whereas it was overestimated in OSE_M_f from 25 September (Supplementary Fig. 7a,c), because the predicted location of Karl was too close to the upper trough relative to that in CTL_1_f (Fig. 2d,g). This resulted in considerable differences not only in the track forecast but also in the predicted central pressure of Karl between CTL_1_f and OSE_M_f. OSE_C_f, which excluded the additional radiosonde observations from the Canadian stations, predicted Karl’s locations to be further north (Fig. 2a). In contrast with OSE_M_f and OSE_C_f, OSE_G_f had large ensemble spreads for both the track and the central pressure (Supplementary Fig. 7a,d). However, differences in the predicted track of Karl and in the upper-troposphere circulations between CTL_1_f and OSE_G_f were very small (Fig. 2a and Supplementary Fig. 2f). Dropsonde observations by Global Hawk reduced the ensemble member spread for predictions of Karl’s location and improved the forecasts of its central pressure. Additional Arctic radiosonde observations also improved the forecasts of Karl’s location.

In contrast to case of Karl, the location of Ian was not captured on 16 September, even in CTL_1_f (Fig. 2b). In addition, neither CTL_1_f nor OSE_M_f captured the temporal evolution of the central pressure of Ian (Supplementary Fig. 8). Although the ACCs of CTL_1_f (OSE_M_f) fell to 0.9 (0.85) and the range of the spread of the ACCs grew from 0.88 (0.82) to 0.95 (0.90) at the 4.0-day forecast, the difference in the ACC of Z300 between CTL_1_f and OSE_M_f was very small with almost the same spread at the 4.5-day forecast (Supplementary Fig. 6b). However, there was a difference in the position of Ian on 18 September between CTL_1_f and OSE_M_f, resulting from differences in the positions of Ian in the merging stage on 16 September (Fig. 2b). OSE_M_ underestimated the southwesterly wind around the trough, which slowed the eastward movement of Ian in the marginal stage (Fig. 2e,h and Supplementary Fig. 3b). Additional Arctic radiosonde observations had an impact on the forecasts of Ian’s location.

We conducted similar experiments initialized by CTL_2_ and OSEs for August (Fig. 2c). In both CTL_2_f and OSE_BAP_f, the centre of TC Lionrock crossed Japan in some of the ensemble forecast members, with strong southerly steering winds at the eastern edge of the trough (Fig. 2f,i). However, in CTL_2_f, the number of members that remained near southern Japan was larger than in OSE_BAP_f. In OSE_BAP_f, overestimated strong southerly winds caused by the trough over the southern Sea of Japan produced an eastward shift compared with CTL_2_f, making the northward movement of the typhoon fast in most members. There were differences in geostrophic wind speed between CTL_2_f and OSE_BAP_f over southern Japan (Supplementary Fig. 4g), resulting from a difference in forecast skill for the trough location at 300 hPa (black contours in Fig. 2f,i and Supplementary Fig. 4g). The ACCs of both CTL_2_f and OSE_BAP_f were <0.8 with large spread (CTL_2_f: from 0.59 to 0.91, OSE_BAP_f: from 0.60 to 0.87) at the 4.0-day forecast and the values remained large with almost the same spread at the 4.5-day forecast as in the previous case, although OSE_BAP_f declined to 0.75 in the 4.5-day forecast (Supplementary Fig. 6c). OSEsf tended to predict an overestimated central pressure of Lionrock at 1200 UTC 29 August compared with CTL_2_f (Supplementary Fig. 9). Errors and uncertainties in the predicted Lionrock tracks were found in other sensitivity experiments (OSE_MID_, _TRO_), which initialized analysis datasets without routine radiosonde observations from operational mid-latitude and tropical stations (Supplementary Fig. 4e,f,k,l; see Methods). In particular, in OSE_MID_f, overestimated southerly winds caused by large errors in upper-troposphere circulations resulted in faster northward movement of Lionrock at 1200 UTC 29 August (Supplementary Fig. 4e,k), revealing that the Arctic radiosonde observations had an impact on the Lionrock track forecast, as did the routine radiosondes at mid-latitudes and in the tropics. To investigate the impact of additional Arctic radiosonde data on the forecast skill of other typhoons over East Asia, we conducted forecast experiments for three typhoons (Chanthu, Mindulle, and Komoasu). In contrast to Lionrock, we did not find an impact of additional Arctic radiosonde observational data on the forecasting of the other typhoons in August 2016, because neither CTL_2_f nor OSE_BAP_f captured the locations of the typhoons over East Asia at the 4.5-day forecast (not shown). In the Lionrock case, CTL_2_f with a small spread of central positions of Lionrock had relatively large values of ACC for Z300 (Fig. 2f,i, and Supplementary Fig. 6c), suggesting that additional Arctic observations had positive impact on the Lionrock track forecast, but intensification was not well forecast in any ensemble.

## Flow-dependent error at upper levels

**Figure 3:**
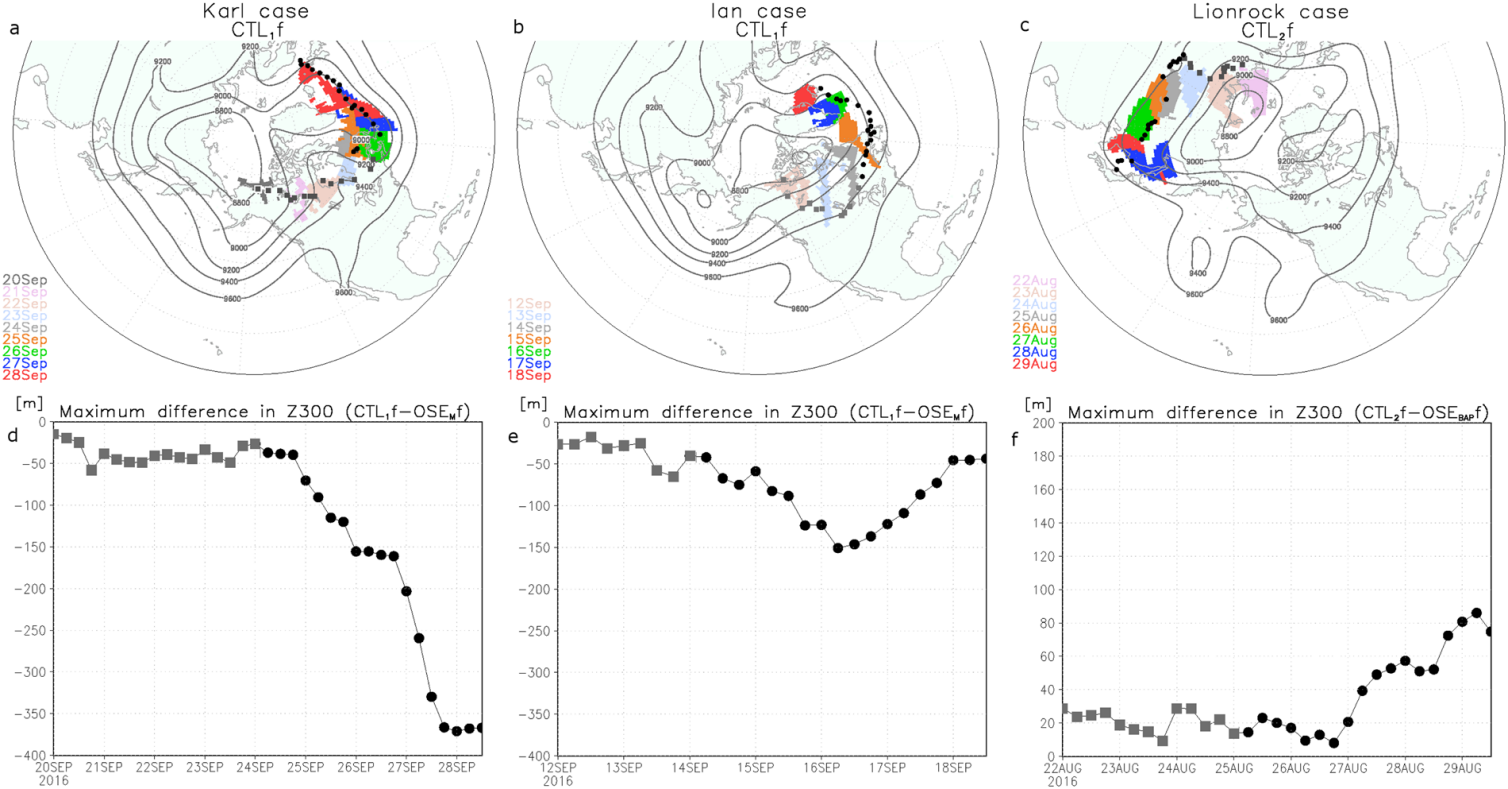
Ensemble mean difference of Z300 between CTLfs and OSEfs and trajectories of potential vorticity (PV) features. (**a**) Time–mean geopotential height on 300-hPa level (Z300: contour interval 200 m) for 24–28 September with regions where PV exceeds 8 PVU on the 330 K surface shown with colour corresponding to location at 0000 UTC on each day (colour shading: PVU) for 20–28 September. (**b**) As in (**a**), but Z300 for 14–18 September with surface where PV exceeds 8 PVU shown with colour corresponding to location at 0000 UTC on each day for 11–18 September. (**c**) As in (**a**), but Z300 for 25–29 August with surface where PV exceeds 4 PVU shown with colour corresponding to location at 0000 UTC on each day for 22–29 August. Some PV fields are masked to highlight temporal evolution of targeted PV. Temporal evolution of maximum value of Z300 difference between CTLfs and OSEfs for Karl case (**d**), Ian case (**e**), and Lionrock case (**f**) before forecast (squares) and during forecast (dots) period. Black squares and dots in (**a**), (**b**), and (**c**) correspond to the location of the maximum Z300 difference at each time. Grid Analysis and Display System (GrADS) version 2.0.2 (http://cola.gmu.edu/grads/) was used to create the maps in this figure.

Based on the experimental results, errors in the predicted upper-level troposphere circulations were considered the source of errors in the motion of Lionrock, Ian and Karl. Atmospheric circulation anomalies associated with blocking^30,31^ and teleconnection patterns^32^ induced large-scale flows from the high latitudes into the mid-latitudes. To understand the origin of the considerable errors in the mid-latitude upper troposphere, it is instructive to track strong PV and the locations of the maximum difference in the ensemble mean Z300 between CTLfs and OSEfs (∆Z300). This is because error and uncertainty are associated with PV along the upper-level isentropic surfaces^33^. In the case of Karl, OSE_M_ had considerable Z300 error associated with a PV feature over the east coast of North America at the initial time (24 September), which can be traced back to near RV *Mirai* on 20 September. The feature moved towards the North Atlantic with strong PV (Fig. 3a). A large ∆Z300 over the Arctic reached the mid-latitudes and maintained large values (~30 m), even in reanalysis fields (Fig. 3d), in both CTL_1_ and OSE_M_. This influenced the prediction of the merging stage of an extratropical cyclone with Karl over the east coast of North America on day 2.0 (Fig. 2a,d,g). In the case of Ian, the considerable ∆Z300 that was near the Chukchi Sea on 12 September remained large and it reached the east coast of North America with strong PV on 14 September (Fig. 3b,e), resulting in differences in the positions of Ian between CTL_1_f and OSE_M_f (Fig. 2b,e,h). In the typhoon case, large ∆Z300 was found over the Barents Sea on 22 August, which crossed central Eurasia until the forecast initiation time (25 August). Finally, the predicted ∆Z300 at 1200 UTC on 29 August reached East Asia (Fig. 3c). In contrast to the previous cases, the ∆Z300 was small, i.e., <20 m at the forecast initiation time (25 August), increasing to at most 70 m by day 4.5 (Fig. 3f), which resulted in smaller differences in the TC tracks between CTLf and OSEf than in the Karl and Ian cases. In general, uncertainty of atmospheric conditions in the initial state became small at mid-latitudes of the Northern Hemisphere because of the relatively large spatial coverage of observation stations. However, large uncertainty sometimes remained considerable in the case of the southward intrusion of strong PV originating over the sparse observing network of the Arctic region (e.g., the Canadian Archipelago) (Fig. 1).

Our results indicate that improvement of forecast upper-troposphere circulations that affect surface circulations sometimes increased the accuracy of TC track forecasts. Therefore, the additional Arctic radiosonde observations, which reduced uncertainty and error for upper-level troposphere circulations at the initial time, improved weather forecasts over the mid-latitudes during summer. Although impacts of the Arctic observations of upper-level troposphere from satellite radiance data can not be assessed in our data assimilation system and should be investigated in the near future, a flow-dependent error propagation associated with a tropospheric polar vortex would be an universal and essential concept from the viewpoint of an observing system design.

The amplitudes of the meandering jet stream (calculated using a “zonal index”) over the Pacific Ocean during August and over the Atlantic Ocean during September 2016 were not the largest during 1979–2016 (not shown). This implies that additional Arctic radiosonde observations sometimes affect the forecasts of tropical and mid-latitude cyclone tracks, even in summer. An increase in the magnitude of the jet stream meander, associated with recent sea ice declines, might increase the frequency of transport of large uncertainty from the Arctic to the mid-latitudes^34^. Although the impact of dropsonde observations has not been evaluated for East Asia in 2016, TC forecasts for East Asia are also influenced by dropsonde data from aircraft^7,8^. Field campaigns scheduled during the Year Of Polar Prediction^35^ and the Years of Maritime Continent from mid-2017 to mid-2019 should provide great opportunity to increase evidence of the effect of additional summer radiosonde observations over the Northern Hemisphere on the predictability of weather extremes at mid-latitudes.

## Methods

### Observations

Supplementary Fig. 1 shows the daily number of radiosonde observations from ships and land stations and of dropsonde observations from the NASA Global Hawk aircraft during August and September 2016. During August, radiosondes were usually launched twice per day, i.e., from RV *Polarstern* at 0600 and 1200 UTC in Fram Strait, and from RV *Araon* at 0000 and 1200 UTC in the Beaufort Sea. During the same period, observations from the Russian Cape Baranova land station were made once per day. Radiosonde observations from RV *Mirai* were made every six hours (0000, 0600, 1200, and 1800 UTC) during September. Over the North Atlantic, the NASA Global Hawk aircraft made dropsonde observations between 20 and 25 September to improve track forecasts of Tropical Storm Karl (Fig. 1). During 18 September and 18 October, the North Atlantic Waveguide and Downstream Impact Experiment increased the number of radiosonde observations at Canadian stations (i.e., Sable Island, Resolute, Iqaluit, Hall Beach, St. John’s west, and Eureka). In addition, routine twice-daily radiosonde observations from three stations at mid-latitudes (Yining, Wulumuqi, and Kuqa) and from three stations at low latitudes (Pangkal Pinang, Jakarta, and Bengkulu), which were upstream of Lionrock with large uncertainties (Fig. 1), were used to compare the impact of additional Arctic observations with those of routine observations at mid-latitudes and in the tropics.

### Data assimilation system (ALEDAS2)

An ensemble data assimilation system called ALEDAS2^36^, which comprises the atmospheric general circulation model for the Earth Simulator (AFES)^37,38^ and local ensemble transform Kalman filter (LETKF)^39^, produced the AFES-LETKF experimental ensemble reanalysis version 2 (ALERA2) dataset. Sixty-three ensemble forecasts were produced with AFES at horizontal resolution T119 (triangular truncation with truncation wave number 119, 1° × 1°) and L48 vertical levels (σ-level, up to ~3 hPa). National Oceanic and Atmospheric Administration daily 0.25° Optimal Interpolation Sea-Surface Temperature version 2 was used for ocean and sea ice boundary conditions^40^. PREPBUFR global observation datasets compiled by the National Centers for Environmental Prediction and archived at the University Corporation for Atmospheric Research, which were assimilated into the ensemble forecast model using the LETKF, were used as observational data. For the OSEs (data denial experiments), two data-assimilation-forecast cycles were run for the periods of August and September 2016. For September, we created CTL_1_, OSE_MGC_, OSE_M_, OSE_G_, and OSE_C_ reanalysis datasets from 15 August to 28 September 2016, and for August, those of CTL_2_, OSE_BAP_, OSE_B_, OSE_A_, OSE_P_, OSE_MID,_ and OSE_TRO_ were created from 26 July to 30 August 2016. These reanalysis datasets were used as initial data for the forecast experiments.

### Forecast experiments

The forecast experiments were performed using AFES as the forecast model, which has the same model description as ALEDAS2. AFES allowed direct comparison of forecast results with the ensemble reanalysis (i.e., CTL). The experiments used ensemble reanalyses CTLs or OSEs for initial values. We conducted 4.5-day integrations in experiments from 0000 UTC 25 August, 0000 UTC 14 September and 0000 UTC 24 September for the Lionrock, Ian and Karl cases, respectively. Synoptic and large-scale circulations in the troposphere and lower stratosphere obtained by ALERA2 are similar to those of other reanalysis products. However, AFES, with its modest horizontal resolution, did not reproduce the TC central pressure as skilfully as other models with relatively higher resolution (Supplementary Fig. 6). In addition, ALERA2 did not assimilate satellite radiance data in the National Centers for Environmental Prediction PREPBUFR. Circles in the figure indicate ensemble spread. Circle centres show the location of the ensemble mean. The circle radius indicates the average difference of the distance between the locations of the ensemble mean and of each member.

## Electronic supplementary material

Supplementary figure
