# Supplementary material for: Impact on predictability of tropical and mid-latitude cyclones by extra Arctic observations
Source: Sci Rep. 2018 Aug 14;8:12104. doi: 10.1038/s41598-018-30594-4 (PMC6092335; doi:10.1038/s41598-018-30594-4)
Supplement: Supplementary file 1 — Supplementary figure [file 41598_2018_30594_MOESM1_ESM.doc]

**Impact on predictability of tropical and mid-latitude cyclones by extra Arctic observations**

**Kazutoshi Sato*,**^1,2^, Jun Inoue^1,3,4^, Akira Yamazaki^3^, Joo-Hong Kim^5^, Alexander Makshtas^6^, Vasilli Kustov^6^, Marion Maturilli^7^, Klaus Dethloff^7^

1: National Institute of Polar Research, Tachikawa, 190-8518, Japan, 2: Kitami Institute of Technology, Kitami, 090-8507, Japan, 3: Application Laboratory, Japan Agency for Marine-Earth Science and Technology, Yokohama, 236-0001, Japan, 4: SOKENDAI (Graduate University for Advanced Studies), Hayama, 240-0193, Japan, 5: Korea Polar Research Institute, Incheon, 21990, Korea, 6: Arctic and Antarctic Research Institute, 199397, Russia, 7: Alfred Wegener Institute, Helmholtz Centre for Polar and Marine Research, Potsdam, 14473, Germany

*Correspondence to: Kazutoshi Sato

Email: [satokazu@mail.kitami-it.ac.jp](mailto:satokazu@mail.kitami-it.ac.jp)

Tel.: +81-157-26-9429

Fax: +81-157-25-8772

**Supplementary Figure 1. Number of daily observations at stations, ships and aircraft**


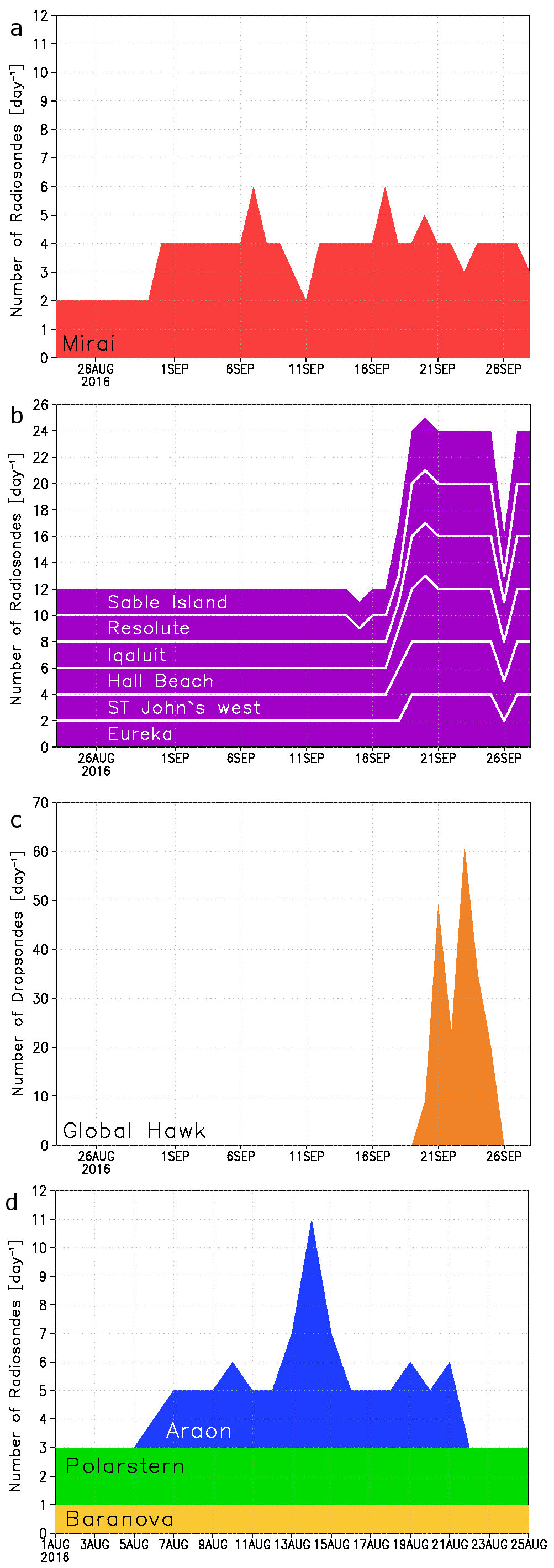


**Supplementary Figure 2 Karl track and upper atmospheric circulation forecast**

Predicted upper level geostrophic wind speed (300–500 hPa), Z300 (black contour) and PV at 330 K (green contour) at 0000 UTC 26 September 2016 (day 2.0 forecast) in CTL_1_ (a), OSE_G_f (b), OSE_C_f (c) and OSE_MGC_f (d). Differences in upper level geostrophic wind speed (shading), Z300 (black contour) and PV on 330 K surface (green line) between CTL_1_f and OSE_M_f (e), OSE_G_f (f), OSE_C_f (g) and OSE_MGC_f (h). Black and orange lines in (a) show TC track from 0000 UTC 13 September through 1200 UTC 29 September in CTL_1_ and ERA-Interim, respectively. Red lines in (b), (c) and (d) show track of Karl from 0000 UTC 24 September through 1200 UTC 28 September in OSE_G_, OSE_C_ and OSE_MGC_, for all ensemble members. Grid Analysis and Display System (GrADS) version 2.0.2 (http://cola.gmu.edu/grads/) was used to create maps in this figure.


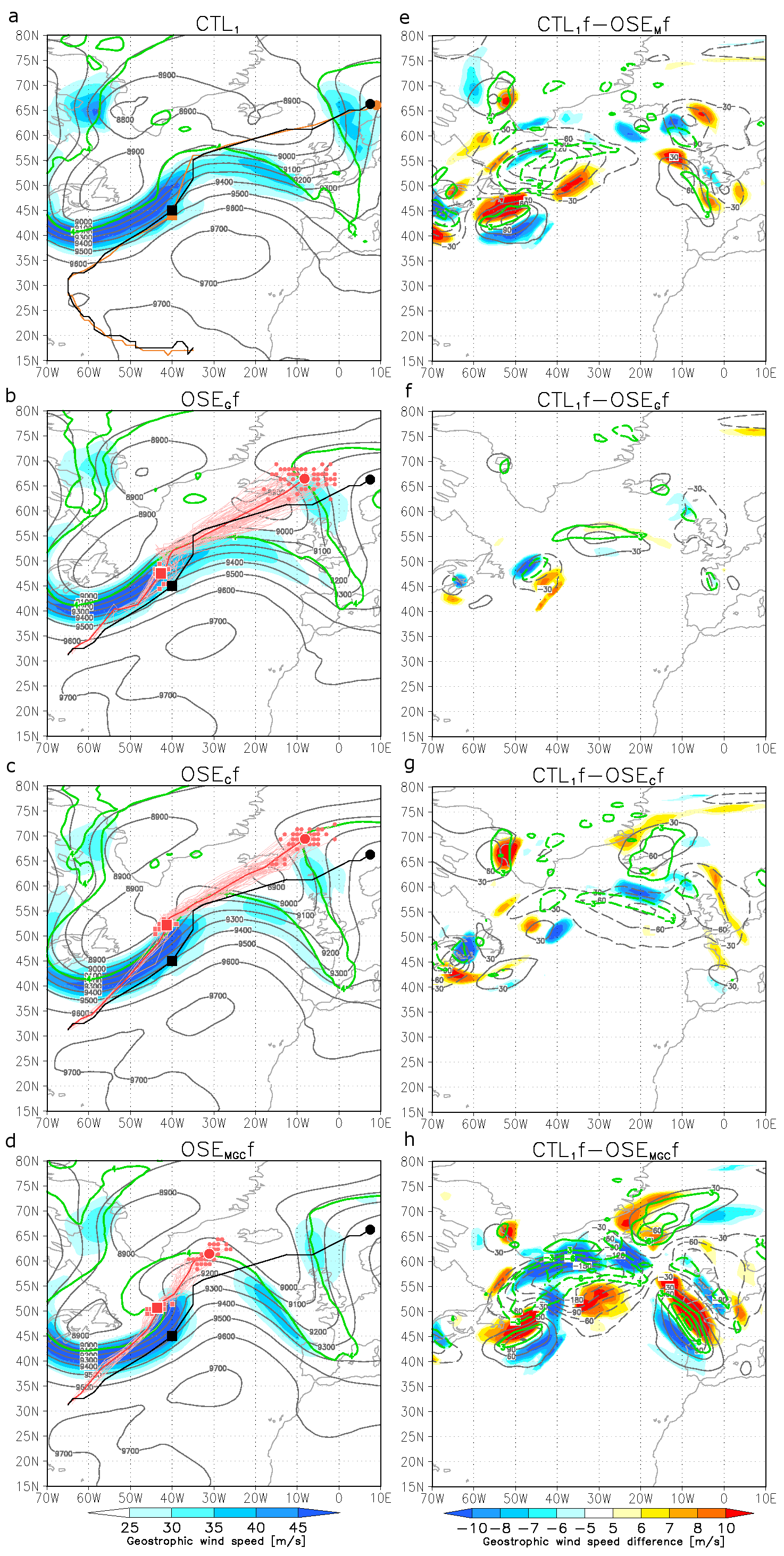


**Supplementary Figure 3 Ian track and upper atmospheric circulation forecast**

a) Upper level geostrophic wind speed (300–500 hPa), Z300 (black contour) and PV at 330 K (green contour) at 0000 UTC 16 September 2016 (day 2.0 forecast) in CTL_1_. (b) Differences in upper level geostrophic wind speed (shading), Z300 (black contour) and PV on 330 K surface (green line) between CTL_1_f and OSE_M_f. Black and orange lines in (a) show TC track from 0000 UTC 12 September through 1200 UTC 18 September in CTL_1_ and ERA-Interim, respectively. Grid Analysis and Display System (GrADS) version 2.0.2 (http://cola.gmu.edu/grads/) was used to create maps in this figure.


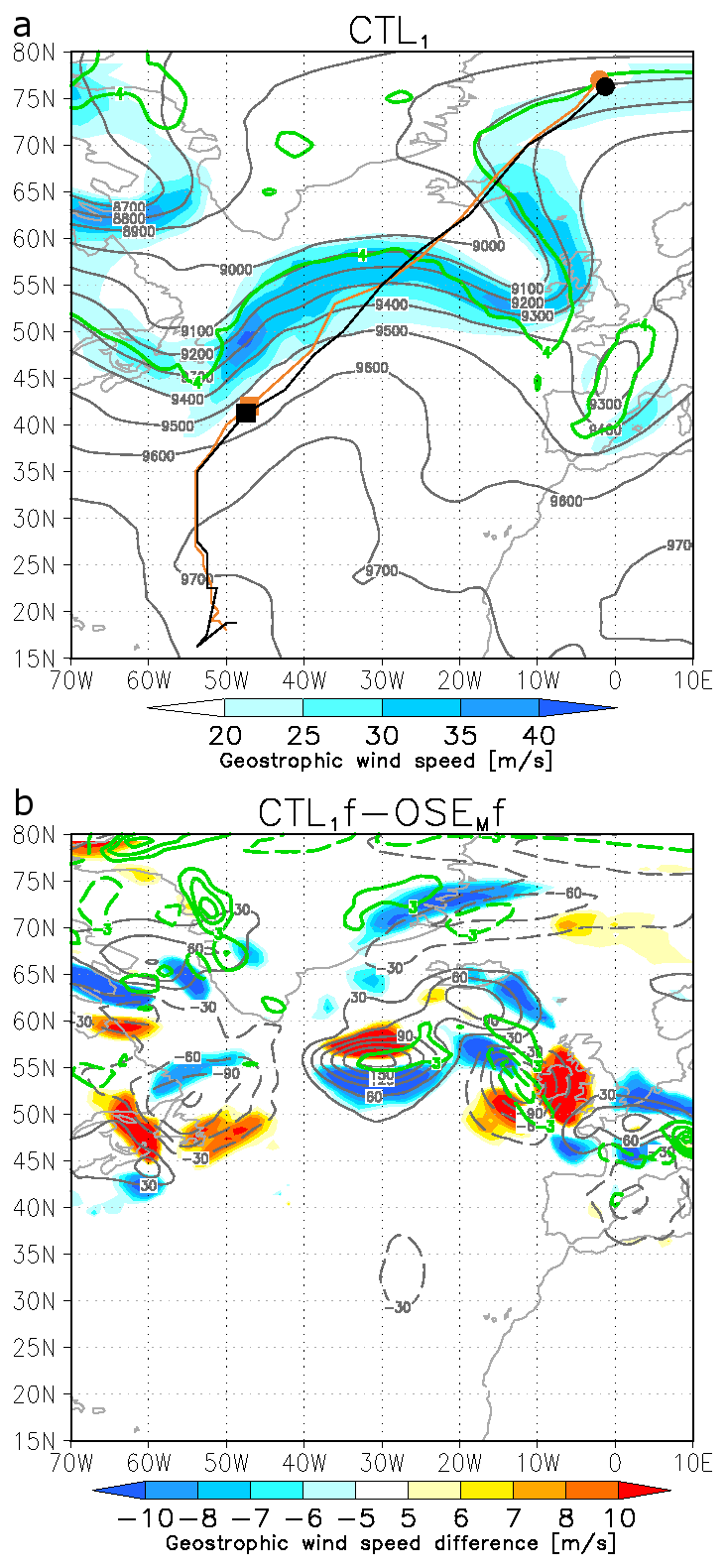


**Supplementary Figure 4 Lionrock track and upper atmospheric circulation forecast**

Predicted upper level geostrophic wind speed (300–500 hPa), Z300 (black contour) and PV at 330 K (green contour) at 1200 UTC 29 August 2016 (day 4.5 forecast) in CTL_2_ (a), OSE_B_f (b), OSE_A_f (c), OSE_P_f (d), OSE_MID_f (e) and OSE_TRO_f (f). Differences in upper level geostrophic wind speed (shading), Z300 (black contour) and PV on 330 K surface (green line) between CTL_1_f and OSE_BAP_f (g), OSE_B_f (h), OSE_A_f (i), OSE_P_f (j), OSE_MID_f (k) and OSE_TRO_f (l). Black and orange lines in (a) show TC track from 1200 UTC 17 August through 1200 UTC 30 August in CTL_2_ and ERA-Interim, respectively. Red lines in (b), (c), (d), (e) and (f) show track of Lionrock from 0000 UTC 24 August through 1200 UTC 29 August in OSE_BAP_, OSE_B_, OSE_A_ OSE_P_ OSE_TRO_ and OSE_MID_ for all ensemble members. Grid Analysis and Display System (GrADS) version 2.0.2 (http://cola.gmu.edu/grads/) was used to create maps in this figure.


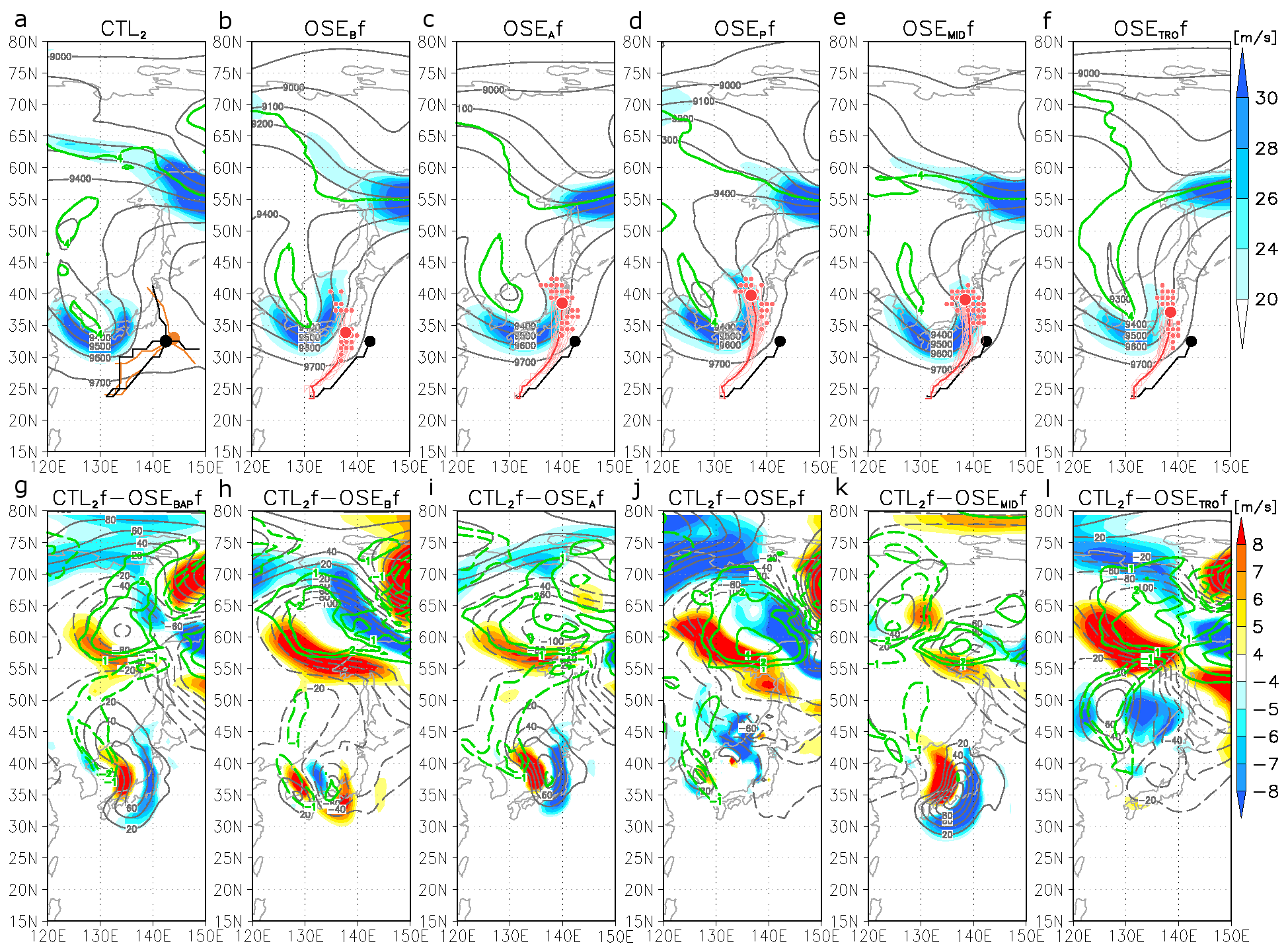


**Supplementary Figure 5 Ensemble difference of Z300 between CTLfs and OSEfs and trajectories of potential vorticity (PV)**

Time-mean geopotential height on 300 hPa level (Z300: conter interval 200 m) during 24 and 28 September with regions where potential vorticity exceeds 8 PVU on 330 K surface shown with color corresponding to location at 0000 UTC on each day (color shading: PVU) during 20 and 28 September in ERA-Interim (a) and CTL_1_ (d). (b), (e) As in (a) and (d), but Z300 during 14 and 18 September with PV exceeds 8 PVU surface shown with color corresponding to location at 0000 UTC on each day during 11 and 18 September. (c), (f) As in (a) and (d), but Z300 during 25 and 29 August with PV exceeds 4 PVU surface shown with color corresponding to location at 0000 UTC on each day during 22 and 29 August. Some PV fields are masked to highlight temporal evolution of targeted PV. Grid Analysis and Display System (GrADS) version 2.0.2 (http://cola.gmu.edu/grads/) was used to create maps in this figure.


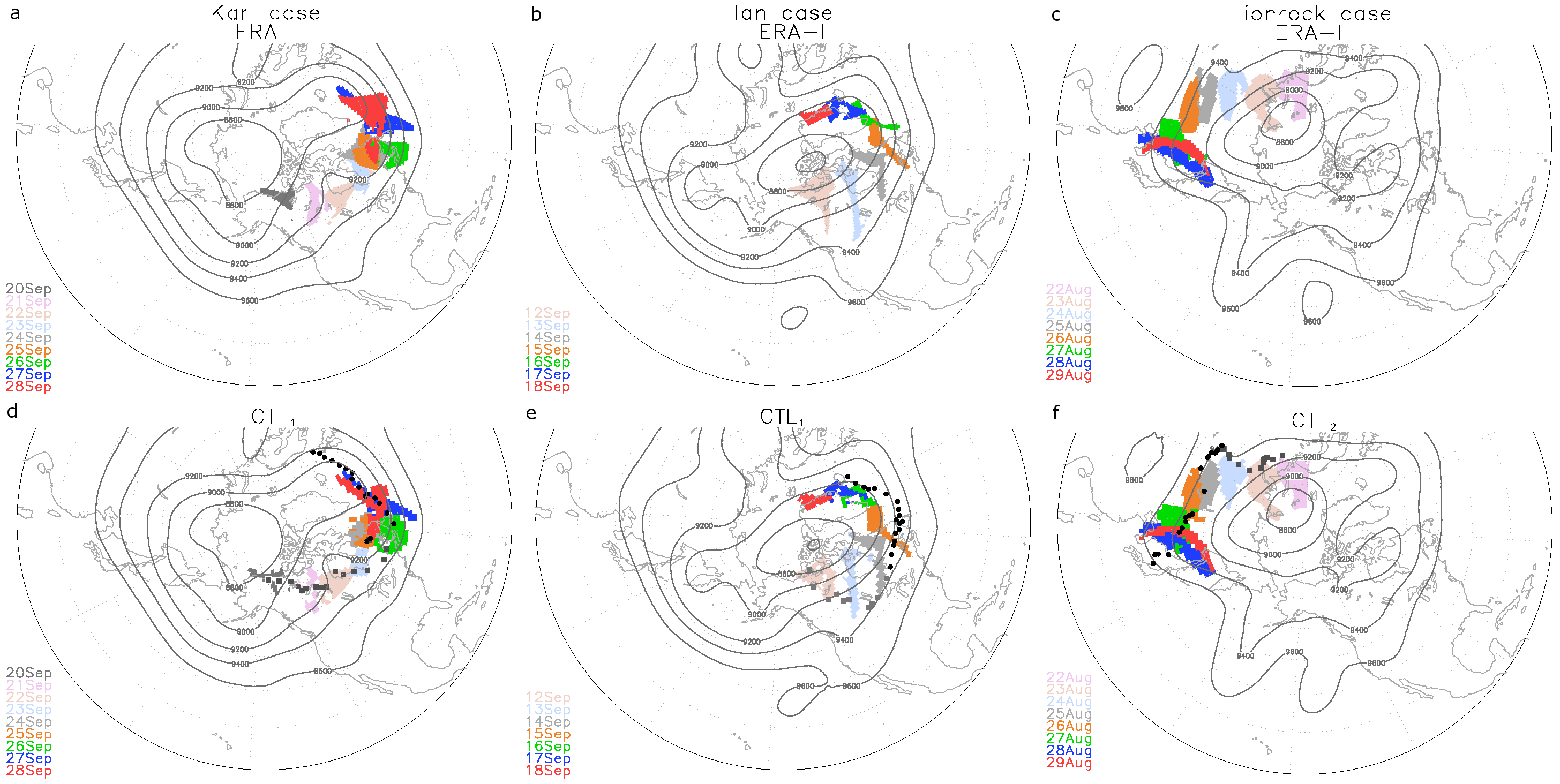


**Supplementary Figure 6 Anomaly correlation coefficients (ACC) over North Atlantic Ocean and East Asia**

**(a) (b)** Temporal evolution of ACC for each ensemble member of CTL_1_f (red lines) and OSE_M_f (blue lines) over North Atlantic Ocean, versus reanalyses. Each thick line shows mean value of ACC. (c) As in a, but each ensemble member of CTL_2_f (red lines) and OSE_BAP_f (blue lines) over East Asia.


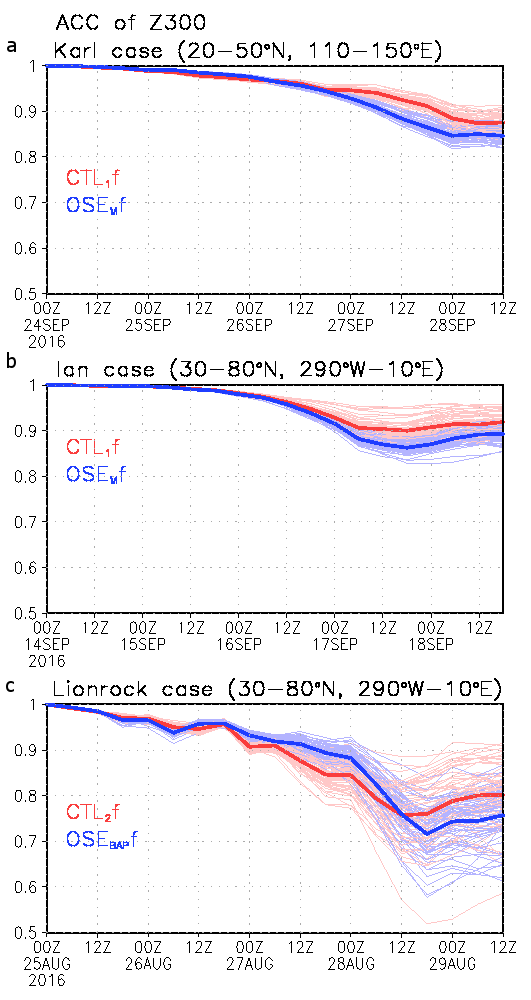


**Supplementary Figure 7 Karl central pressure forecast**

Predicted mean central pressure of Karl in ERA-Interim, CTL_1_, CTL_1_f, OSE_M_f, OSE_G_f, OSE_C_f and OSE_MGC_f (a), and predicted central pressure for each ensemble member of CTL_1_f (b), OSE_M_f (c), OSE_G_f (d), OSE_C_f (e) and OSE_MGC_f (f). Thin and thick colored lines show temporal evolution of central pressure for each member and mean. Thick black lines show temporal evolution of central pressure in CTL_1_.


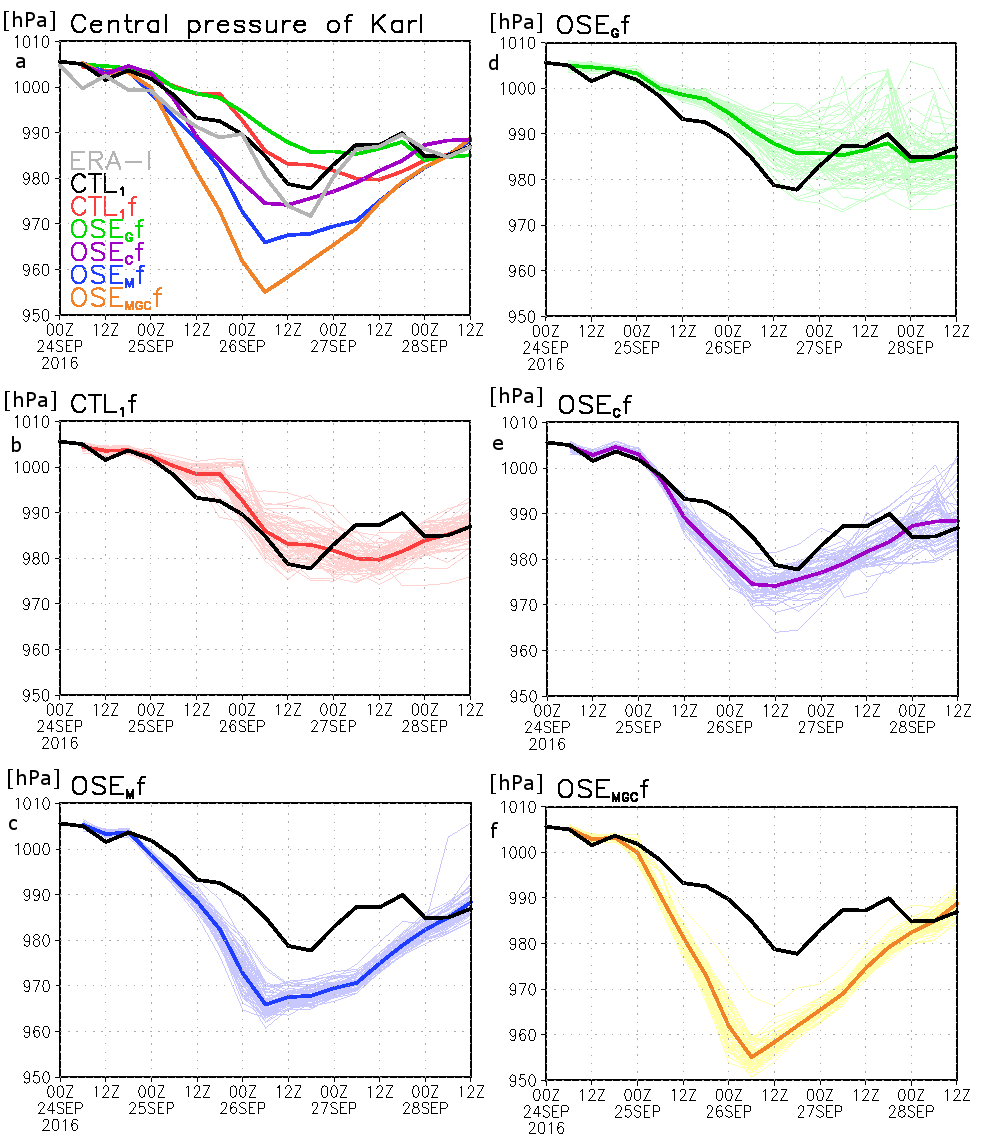


**Supplementary Figure 8 Ian central pressure forecast**

Predicted mean central pressure of Ian in ERA-Interim, CTL_1_, CTL_1_f, OSE_M_f (a), and predicted central pressure for each ensemble member of CTL_1_f (b), OSE_M_f (c). Thin and thick colored lines show temporal evolution of central pressure for each member and mean. Thick black lines show temporal evolution of central pressure in CTL_1_.


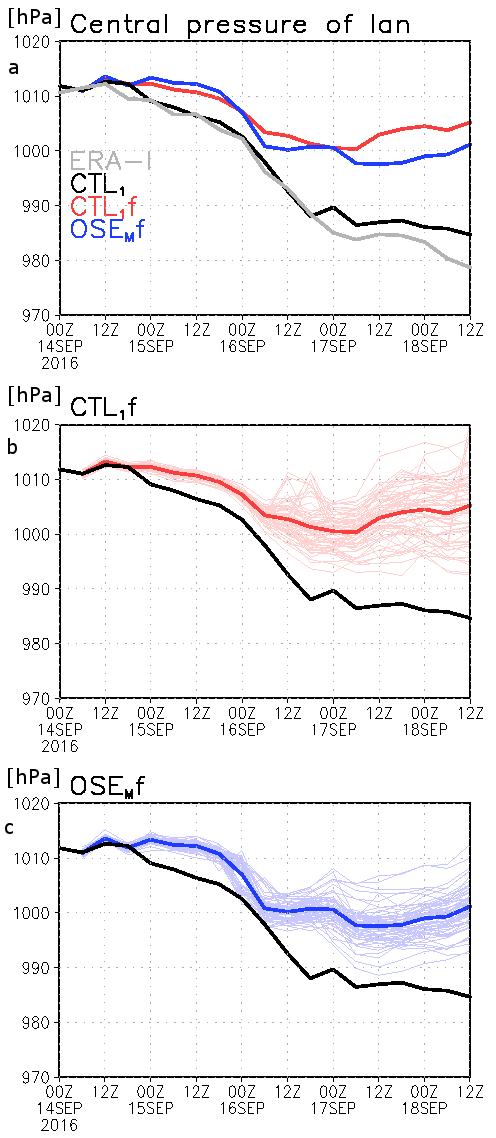

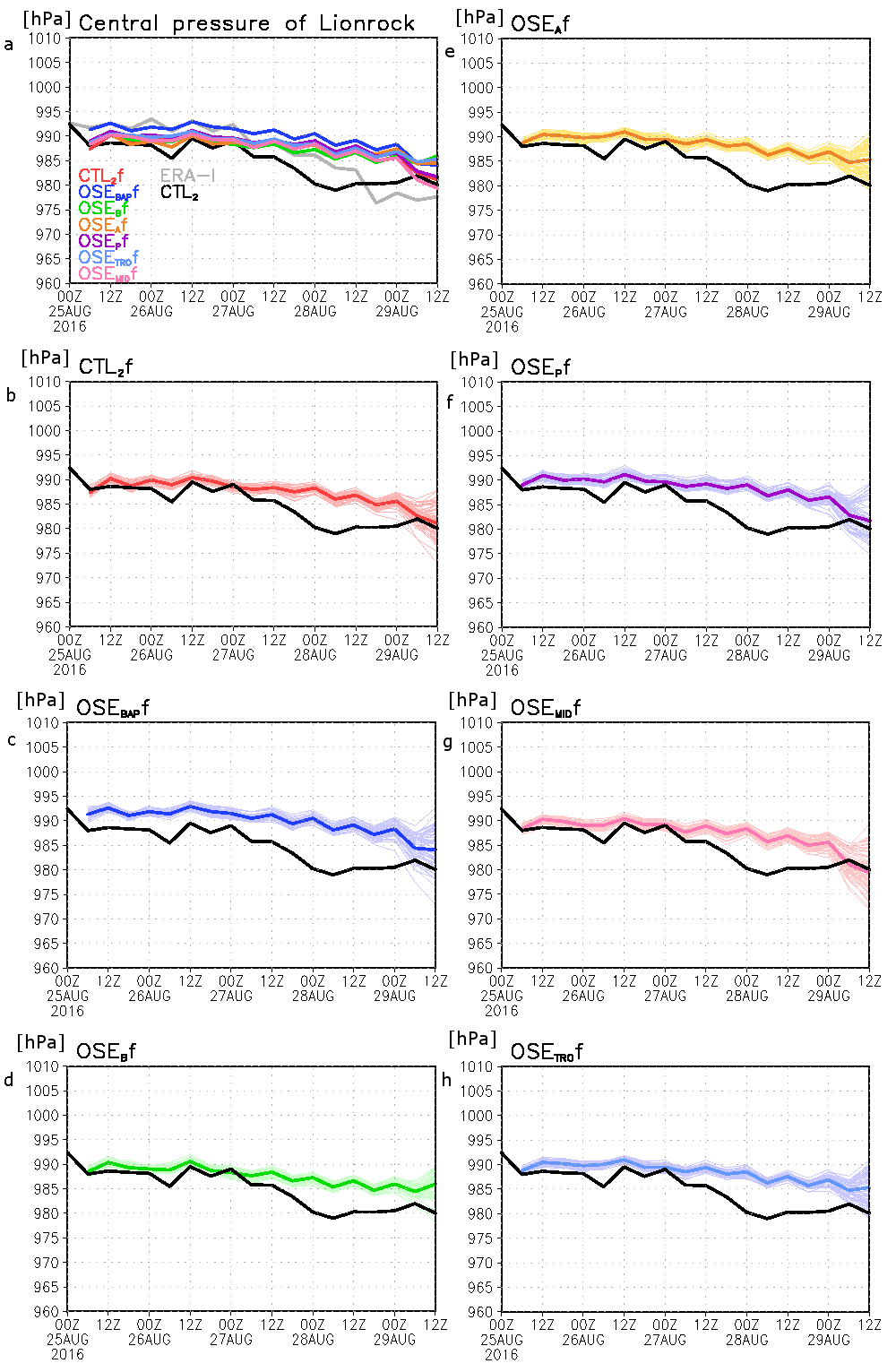


**Supplementary Figure 9 Lionrock central pressure forecast**

Predicted mean central pressure of Lionrock in ERA-Interim, CTL_2_, CTL_2_f, OSE_BAP_f, OSE_B_f, OSE_A_f, OSE_P_f, OSE_MID_f and OSE_TRO_f (a), and predicted central pressure for each ensemble member of CTL_1_f (b), OSE_BAP_f (c), OSE_B_f (d), OSE_A_f (e), OSE_P_f (f), OSE_MID_f (g) and OSE_TRO_f (h). Thin and thick colored lines show temporal evolution of central pressure for each member and mean. Thick black lines show temporal evolution of central pressure in CTL_2_.
